# Gastrointestinal response to the early administration of antimicrobial agents in growing turkeys infected with *Escherichia coli*

**DOI:** 10.1016/j.psj.2024.103720

**Published:** 2024-04-03

**Authors:** Dariusz Mikulski, Jerzy Juśkiewicz, Katarzyna Ognik, Bartosz Fotschki, Bartłomiej Tykałowski, Jan Jankowski

**Affiliations:** ⁎ Department of Poultry Science and Apiculture, University of Warmia and Mazury in Olsztyn, Olsztyn 10-719, Poland; † Institute of Animal Reproduction and Food Research of Polish Academy of Sciences, 10-748 Olsztyn, Poland; ‡ Department of Biochemistry and Toxicology, University of Life Sciences, Lublin 20-950, Poland; § Department of Poultry Diseases, Faculty of Veterinary Medicine, University of Warmia and Mazury in Olsztyn, Olsztyn 10-719, Poland

**Keywords:** antibiotic, avian pathogenic *Escherichia coli*, gastrointestinal tract, turkey

## Abstract

This study investigated the effects of the early administration of enrofloxacin (**E**) or doxycycline (**D**) for the first 5 consecutive days of life, or the continuous administration of the coccidiostat monensin (**M**) throughout the rearing period on gastrointestinal function in turkeys infected with avian pathogenic *Escherichia coli* (**APEC**) in an early or later stage of rearing. Experiment 1 lasted 21 d, and turkeys in groups E, D, and M were infected with APEC on d 15. Experiment 2 lasted 56 d, and it had a factorial arrangement of treatments where birds in groups E, D, and M were infected with APEC on d 15 or d 50. In both experiments, control groups (**C**) consisted of infected and uninfected birds without antibiotic or coccidiostat administration. On d 21 (Experiment 1) and d 56 (Experiment 2), 8 birds from each subgroup were killed, and the ileal and cecal digesta were sampled to analyze the activity of bacterial enzymes and the concentrations of short-chain fatty acids (**SCFA**). The experimental treatments did not affect the final body weight or body weight gain of birds. Both experiments demonstrated that APEC contributed to an increase in ammonia levels of the cecal digesta (means from 2 experiments: 0.311 vs. 0.225 mg/g in uninfected birds) and ileal pH (6.79 vs. 6.00) and viscosity (2.43 vs. 1.83 mPa⋅s). Moreover, the *E. coli* challenge enhanced the extracellular activity of several cecal bacterial enzymes, especially in older turkeys infected with APEC in a later stage of life. The continuous administration of monensin throughout the rearing period resulted in a weaker gastrointestinal response in older birds, compared with the other 2 antibiotics administered for the first 5 d of life. The results of the study are inconclusive as both desirable and undesirable effects of preventive early short-term antibiotic therapy were observed in turkeys, including normalization of ileal viscosity and cecal ammonia concentration (positive effect), and disruption in cecal SCFA production (negative effect).

## INTRODUCTION

Antimicrobial agents play a key role in the prevention and treatment of diseases in poultry production. Avian colibacillosis poses a significant threat in poultry production around the world because it leads to premature death, decreases productivity, and compromises bird welfare (De Oliveira et al., 2020; Alber et al., 2021). Colibacillosis is caused by avian pathogenic *Escherichia coli* (**APEC**) which are most frequently isolated from birds, in particular from respiratory and reproductive systems. APEC can act as both primary and secondary pathogens that infect birds of all ages (Dho-Moulin and Fairbrother, 1999; Dziva and Stevens, 2008). Infections tend to be more severe in young birds, where the mortality rate can be as high as 20% (Dho-Moulin and Fairbrother, 1999; Swelum et al., 2021). Avian colibacillosis can induce various clinical symptoms, including respiratory symptoms associated with respiratory tract infections, decreased egg production associated with reproductive tract infections, severe depression, and sudden death caused by septic shock with characteristic anatomopathological changes such as perihepatitis, pericarditis, and airsacculitis (Sadeyen et al., 2014). In commercial broiler chicken and turkey farms, *E. coli* infections are managed with antibiotics, phytoncides, and vaccines (Huff et al., 2004; Velkers et al., 2005; Garmyn et al., 2009; Li et al., 2015; Śmiałek et al., 2020). Excessive and uncontrolled use of antibiotics raises serious concerns due to growing levels of antimicrobial resistance (Ceccarelli et al., 2020; Kim et al., 2020; Christensen et al., 2021).

To date, the efficacy of antibiotics in the treatment of poultry diseases has been evaluated based on the results of clinical trials, microbiological tests, and analyses of histopathological lesions (Cagnardi et al., 2014; Zhuang et al., 2014; Trouchon and Lefebvre, 2016; Grabowski et al., 2022). “Preventive” administration of low antibiotic doses can induce undesirable changes in the pharmacokinetics of antimicrobials in the tissues of infected poultry (Manyi-Loh et al., 2018). Research has shown that enrofloxacin can exert hepatotoxic effects, alter the activity of liver enzymes, induce inflammation, and suppress the immune response (Grabowski et al., 2022).

Most studies analyzing the mechanism of action of enrofloxacin, monensin, and doxycycline in animals focused on changes in the species composition of gut microbiota, rather than their metabolic activity, including the production of short-chain fatty acids (**SCFAs**) (Ma et al., 2020; Vieira et al., 2020; Greene et al., 2022). Bacteriological examinations (e.g., identification of selected bacterial groups) are a useful tool for describing the gut ecosystem, but they have limited applicability in studies of metabolism, nutrition and the health status of birds (Aruwa et al., 2021). An alternative approach is to perform biochemical assays that measure the functional activity of the entire microbiome. Their results can be used to determine the role played by the microbial community in intestinal metabolism. Therefore, attempts were made in previous studies to analyze the content and profile of bacterial fermentation end-products (ammonia, SCFAs) as well as the activity of selected bacterial enzymes in the large intestinal digesta, in the latter case instead of bacterial counts (Zduńczyk et al., 2014; Przywitowski et al., 2017). According to Cuccato et al. (2022), antibiotics can compromise the intestinal health of broilers by increasing intestinal permeability, promoting inflammatory responses, and damaging the intestinal epithelium. In turn, Boynton et al. (2017) and Greene et al. (2022) demonstrated that even short-term early administration of doxycycline to noninfected broiler chickens and healthy growing mice induced long-term changes in the composition of cecal microbiota.

In our previous study, the enzyme activity of cecal microbiota tended to decrease in clinically healthy turkeys receiving enrofloxacin or doxycycline (Mikulski et al., 2022). However, little is known about the potential risks associated with the early administration of antibiotics to healthy birds or birds infected with APEC, including intensified oxidative stress, changes in the composition of cecal microbiota, and the metabolic activity of the gastrointestinal tract (**GIT**).

This study presents the results of 2 experiments conducted on growing turkeys. The aim of these experiments was to determine whether the early administration of an antibiotic (enrofloxacin or doxycycline) or a diet containing the coccidiostat monensin affect the metabolic activity of cecal microbiota in turkeys infected with APEC. The objective of Experiment 1, which lasted 21 d, was to describe the changes that were expected to occur in young birds challenged with APEC on d 15, that is, 6 d before the completion of the study. It was hypothesized that the early administration of enrofloxacin (**E**) or doxycycline (**D**) for the first 5 consecutive days, or the continuous administration of the coccidiostat monensin (**M**) during the entire rearing period (d 0–21) would affect the activity of cecal microbial enzymes and the production of SCFAs in turkeys infected with APEC on d 15. In Experiment 2, the antibiotic treatment protocol was similar to that applied in Experiment 1 (E and D were administered for the first 5 consecutive days of feeding), and M was administered during the entire rearing period, d 0–56), and the activity of cecal microbial enzymes and SCFA production were examined at the completion of the study on d 56 in turkeys infected with APEC on d 15 or d 50 (early or late infection, respectively). In older turkeys, it was hypothesized that a different date of inducing APEC infection, that is, in an early or later stage of rearing, would differently affect the activity of cecal microbial enzymes and the production of SCFAs.

## MATERIALS AND METHODS

This study is part of multiaspect experiments conducted by 2 research teams. Selected findings from this project, including gastrointestinal function, inflammatory responses and the morphological structure of selected organs of the immune system and redox status in the blood serum of turkeys treated with antibiotics, have already been published in thematically linked articles (Mikulski et al., 2022; Nadolna et al., 2023; Smagieł et al., 2023a, 2023b).

### Ethics Statement

The experiment was conducted in the Animal Research Laboratory of the Department of Poultry Science and Apiculture, University of Warmia and Mazury in Olsztyn (Poland). The study protocol was approved by the Local Ethics Committee for Animal Experiments (resolution No. 47/2021), and the animals were cared for under guidelines comparable to those laid down by EU Directive 2010/63/EU.

### Birds, Housing, and Diets

Samples of ileal and cecal digesta were obtained from female Hybrid Converter Novo turkeys fed experimental diets for 21 d and 56 d. A total of 200 Hybrid Converter Novo female turkeys were placed in 4 pens on litter (wood shavings, 4 m^2^ each) on the day of hatch, and were randomly assigned to 4 experimental groups (C, M, E, D), with 50 birds per treatment. The average initial d 0 body weight (**BW**) of poults was 56.9 g (SD = 0.254). On d 7, all birds were individually marked with numbered wing tags. Group E received enrofloxacin (Enrofloxacin 10%, Biowet, Drwalew, Poland), group D received doxycycline (Doxylin CT WSP 433 mg/g, Dopharma Research B.V., Raamsdonksveer, Netherlands), and group M was administered the coccidiostat monensin (Coxidin 200, Huvepharma Polska, Warsaw, Poland). The antibiotics enrofloxacin and doxycycline were added to drinking water for 5 consecutive days after hatching (the EU authorized dose of 10 mg enrofloxacin/kg BW and 50 mg doxycycline/kg BW, daily). Monensin was added to feed continuously for the entire rearing period (90 mg/kg, for 56 d). Control C group birds were not administered any antibiotics or the coccidiostat.

The conditions of the room were maintained according to standard management practices in a commercial turkey house (Hybrid Turkeys, 2020). The microclimate in the housing facility was controlled automatically, and the conditions were adjusted to the birds’ age. All birds were fed isocaloric diets, which met their nutrient requirements according to the nutrient guidelines for commercial turkeys (Hybrid Turkeys, 2020). The feed was given as crumbles from d 0 to d 28, and as pellets from d 29 to d 56. Throughout the rearing period, the birds had unlimited access to feed and water. The composition and nutrient content of diets are presented in Supplementary Table 1.

### Experimental Design and Number of Birds

Two experiments were performed on turkeys that were reared until 56 d of age. Experiment 1 lasted 21 d, and it involved a 4  ×  2 factorial arrangement of antibiotic (E, D, M, or C) administration and challenge with APEC (no challenge or challenge on d 15) in a completely randomized design. At d 15, 19 birds from each of groups C, M, E, D (76 birds in total) were moved to a separate part of the building, and were infected with APEC (C+, M+, E+ D+). On d 21, 8 birds from each of noninfected groups C-, M-, E-, D- and 8 infected birds from groups C+, M+, E+, D+ were slaughtered. The remaining 11 birds from each group of infected birds were used in Experiment 2 (early R challenge).

In Experiment 2, the antibiotic treatment protocol was similar to that applied in Experiment 1 (E and D were administered for the first 5 d of feeding, and M was administered during the entire rearing period of 56 d). Experiment 2 involved a 4  ×  3 arrangement of antibiotic (E, D, M, or C) administration and challenge with APEC (no challenge or challenge on d 15 or challenge on d 50; marked as N or R or L, respectively) in a completely randomized design. Thus, turkeys were divided into 12 groups in Experiment 2: CN (control, no antibiotics, uninfected), MN (monensin, uninfected), EN (enrofloxacin, uninfected), DN (doxycycline, uninfected), and CR, MR, ER, DR as well as CL, ML, EL, DL (as in the above groups but with experimental early, R or late L, infection with APEC, on d 15 and d 50, respectively). On d 50, 11 turkeys from each of groups C, M, E, D (44 birds in total) were moved to a separate part of the building, and were infected with APEC (CL, ML, EL, DL). On d 56, 8 birds from groups CN, MN, EN, DN not infected with APEC, 8 birds from groups CR, MR, ER, DR infected on d 15, and 8 birds from groups CL, ML, EL, DL infected on d 50 were slaughtered.

In both experiments, 3 additional birds were included in the groups of uninfected and infected turkeys to replace dead birds. Mortality cases due to infection were not recorded, and the unused additional turkeys, including 12 birds infected on d 15 and 12 birds infected on d 50, were disposed of at the end of the experiment.

### Experimental Infection With APEC

On d 15 and d 50, 8 turkeys were randomly selected from each uninfected group and each group infected with APEC, according to the method described by Mazur-Gonkowska et al. (2004). In Experiment 1 (completed on d 21), infection was induced on d 15, and the infected groups are marked in the Tables with the “+” sign, and the uninfected groups are marked with the “-” sign (C+, M+, E+, D+ and C-, M-, E-, D-, respectively). In Experiment 2 (completed on d 56), infection was induced on d 15 (R, early infection) and on d 50 (L, late infection), and the groups are marked in the Tables as CR, MR, ER, DR (early infection on d 15), CL, ML, EL, DL (late infection on d 50) and CN, MN, EN, DN (uninfected groups). In brief, APEC [*E. coli* O78: K80; virulence factors: *cvi/cva+, vat-, tsh+, iucD+, papC+, irp2+, iss+, astA+*; received from the RB VAC veterinary laboratory (Zielona Góra, Poland)] was injected into the left abdominal air sac at a dose of 4 × 10^5^ colony-forming units (**CFU**) per turkey in 0.3 mL of 0.9% NaCl solution. Uninfected birds received 0.3 mL of sterile 0.9% NaCl solution into the abdominal air sac at the same time and via the same route. Birds infected with APEC and uninfected birds were kept in group pens in separate sections of the building, and were handled by different employees to prevent cross-contamination.

### Sample Collection

All birds were weighed individually on d 15 and d 50, and before slaughter on d 21 and d 56. Body weight gains were determined based on individual BW of birds slaughtered on d 21 and d 56. At the end of the evaluation period, on d 21 (Experiment 1) and d 56 (Experiment 2), 8 birds from each group were weighed and euthanized, and samples were collected to determine gut physiology parameters. Birds were sacrificed by decapitation after electrical stunning. The following parameters were assessed: small intestine (pH of ileal digesta, dry matter (**DM**) percentage of ileal digesta, ileal digesta viscosity), ceca (pH of digesta, DM percentage of digesta, ammonia concentration, extracellular, intracellular and total activities of selected bacterial enzymes, SCFA concentration, bile acid concentration).

### Brief Description of Analytical Procedures

#### Dry Matter Analysis

Fresh samples of ileal (middle section of the ileum) and cecal digesta were used for immediate analysis, and the remaining samples were transferred into sterile tubes, frozen in liquid N, and stored at −70°C. The DM content of ileal and cecal digesta was determined at 105°C. In fresh cecal digesta, ammonia was extracted, trapped in a solution of boric acid in Conway's dishes, and determined by direct titration with sulfuric acid (Hofirek and Haas, 2001). The ileal digesta was vortexed and centrifuged at 7,211 *g* for 10 min. The supernatant fraction (0.5 mL) was placed in the Brookfield LVDV-II+ cone-plate rotational viscometer (CP40; Brookfield Engineering Laboratories, Stoughton, MA), and viscosity was measured at a constant temperature of 397°C and a shear rate of 60/s. Viscosity was recorded as apparent viscosity.

### Enzyme Activity

The activity of gut microbiota was measured based on the activity of bacterial enzymes and the concentrations of SCFAs. Bacterial enzyme activity in the cecal digesta was determined spectrophotometrically based on the rate of p- or o-nitrophenol release from their respective nitrophenylglucosides. The extracellular activity of selected cecal bacterial enzymes (α- and β-glucosidase, α- and β-galactosidase, β-glucuronidase, α-arabinopyranosidase, β-xylosidase) released out of bacterial cells into the intestinal digesta was measured with the aid of appropriate nitrophenylglucosides (Juśkiewicz and Zduńczyk, 2002). After incubation (39°C) of the substrate and digesta solutions in 100 mM phosphate buffer (pH 7.0), p-nitrophenol and o-nitrophenol concentrations were quantified at 400 nm and 420 nm, respectively. Enzyme activity was expressed as µmol of the product formed per h per g of digesta. The following substrates were used (SIGMA, Poznań, Poland): p-nitrophenyl-α-D-glucopyranoside (for α-glucosidase), p-nitrophenyl-β-D-glucopyranoside (for β-glucosidase), p-nitrophenyl-α-D-galactopyranoside (for α-galactosidase), o-nitrophenyl-β-D-galactopyranoside (for β-galactosidase), p-nitrophenyl-β-D-glucuronide (for β-glucuronidase), p-nitrophenyl-α-L-arabinopyranoside (for α-arabinopyranosidase), p-nitrophenyl-β-D-xylopyranoside (for β-xylosidase). In order to measure the activity of enzymes secreted by bacterial cells into the cecal environment (extracellular activity), a reaction mixture was prepared containing 0.3 mL of a substrate solution (5 mM) and 0.2 mL of a 1:10 (v/v) dilution of the cecal sample in 100mM phosphate buffer (pH 7.0) after centrifugation at 7,211 *g* for 15 min. Incubation was carried out at 39°C, p-nitrophenol was quantified at 400 nm and o-nitrophenol was quantified at 420 nm after the addition of 2.5 mL of 0.25 M-cold sodium carbonate. Enzyme activity was expressed as μmol product formed per hour per g of digesta. In order to determine the total activity of selected cecal bacterial enzymes, including extracellular activity (see the procedure above) and intracellular activity, a cecal digesta sample diluted in phosphate buffer was mechanically disrupted by vortexing with glass beads (212–300 μm in diameter; 4 periods of 1 min with 1 min cooling intervals on ice) using the FastPrep-24 homogenizer (MP Biomedicals, Santa Ana, CA). The resulting mixture was centrifuged at 7,211 *g* for 15 min at 4°C. The supernatant was used for the enzyme assay described above. Intracellular enzyme activity was calculated by comparing total enzyme activity with the activities of bacterial enzymes secreted into the intestinal environment, and it was expressed as μmol product (**PNP** or **ONP**, p-nitrophenol or o-nitrophenol, respectively) formed per hour per g of digesta. In order to prepare the calculation formulas, the model curves for PNP and ONP (PNP or ONP standard solution in a 100 mM phosphate buffer pH 7.0, 40 mg/L) were used and appropriate equations were obtained. Extracellular enzyme activity was also calculated as the rate of enzyme release, expressed as a percentage of total enzyme activity. All analyses were performed in duplicate.

### Short-Chain Fatty Acid Production

Cecal SCFA concentrations were analyzed by gas chromatography (Shimadzu GC-2010, Shimadzu, Kyoto, Japan) as described previously (Jankowski et al., 2013). The samples (0.2 g) were mixed with 0.2 mL of formic acid, diluted with deionized water, and centrifuged at 7,211 *g* for 10 min. The supernatant was transferred to a vial and loaded onto a capillary column (SGE BP21, 30 m × 0.53 mm) using an on-column injector. Initial oven temperature was 85°C, it was raised to 180°C in steps of 8°C/min, and maintained for 3 min. The temperature of the flame ionization detector and the injection port was 180°C and 85°C, respectively. The volume of the sample for gas chromatography was 1 µL. The concentrations of cecal/colonic putrefactive SCFAs (**PSCFAs**) were calculated as the sum of iso-butyrate, iso-valerate and valerate in the digesta. All SCFA analyses were performed in duplicate. Pure acetic, propionic, butyric, iso-butyric, iso-valeric and valeric acids were obtained from Sigma (Poznan, Poland), and they were combined to create a standard plot and calculate the amount of each acid. An additional set of pure acids was included in each GC run at 5 sampling intervals to maintain calibration.

### Bile Acid Concentration

Bile acids in the cecal digesta were assessed using a Shimadzu LC system (Kyoto, Japan) coupled to the Shimadzu LCMS-2020 mass spectrometer (Kyoto, Japan). Chromatographic separation was conducted using an Avantor ACE, C18-amide, 75 × 2.1 mm, 2.7-µm column by gradient elution with 0.01% (v/v) formic acid in water (solvent A) and a mixture of methanol and acetonitrile in the 1:9 ratio (v/v) (solvent B). The column temperature was set at 45°C, the flow rate was 0.35 mL/min, and the gradient program was as follows: 0 to 8 min, 40% (v/v) B; 8 to 14 min, 50 % (v/v) B; 14 to 15.3 min, 50 - 100% (v/v) B; 15.3 to 17.4 min, 40% (v/v). The injection volume was 1 µL. Mass data acquisitions were performed using LabSolutions 5.109 software (Shimadzu, Kyoto, Japan). A quantitative analysis was carried out with external standards (0.01–1 μM) that had been used to construct linear calibration curves with correlation coefficients of 0.994–0.997. All analyses were performed in triplicate for each sample.

### Statistical Analysis

The data were subjected to 2-way ANOVA to examine the following effects: 1) main effect of antibiotic (A) (C, M, E, D); 2) main effect of challenge (**Ch**) with APEC (in Experiment 1: no challenge or challenge on d 15, and in Experiment 2: no challenge or challenge on d 15 or challenge on d 50); and 3) interaction between the antibiotic factor and the challenge factor (A × Ch). All data were analyzed using the general linear model (**GLM**) procedure of STATISTICA software version 13.1 (TIBCO Inc., Palo Alto, CA). When a significant interaction effect was noted, Tukey's test was used to determine differences between the experimental factors. Data variability was expressed as pooled standard errors of the mean (**SEM**), and *P* < 0.05 was considered statistically significant.

## RESULTS

Neither antibiotic administration nor the APEC challenge applied in an early or later stage of rearing significantly affected the body weight gain of turkeys [Supplementary Tables 2 and 3; Experiment 1 and 2, respectively]. Furthermore, none of the antibiotic-treated groups differed significantly in BW from the untreated, infected control group. No cases of mortality due to APEC infection were recorded in any of the experiments. In all treatment and control groups, the small intestine and ceca had normal morphology. After APEC infection, turkeys showed increased thirst, decreased mobility, and gloominess. They had ruffled feathers for 2 to 3 d, and some of them sneezed after increased physical activity. When samples of internal organs were collected from slaughtered turkeys, turbidity and thickening of the air sac wall (where the inoculum was administered during the experimental infection with APEC) were observed.

### Experiment 1

Two-way ANOVA revealed an A × Ch interaction for DM concentration, viscosity, and pH in the ileal digesta of young turkeys at 21 d of age (Table 1). Ileal DM concentration was highest in group C- (uninfected control turkeys) and lowest in M+ birds (*P* < 0.05 vs. C+, M+, D- and *P* < 0.05 vs. C-, M-, E-, D+, respectively). Additionally, the challenge with *E. coli* reduced ileal DM concentration between C (C+ < C-) and M (M+ < M-) counterparts. Ileal viscosity was highest in groups C+ and M+ (*P* < 0.05 vs. D+). The pH of ileal digesta was highest in group M+ (*P* < 0.05 vs. C-, E-, D-, D+) and lowest in C- birds (*P* < 0.05 vs. C+, M+, E+). As indicated by a significant A × Ch interaction, cecal ammonia concentration was highest in group D+ (*P* < 0.05 vs. all groups except C+, M+). All uninfected birds had significantly lower cecal ammonia levels than C+ and D+ turkeys (*P* < 0.05). The challenged turkeys treated with doxycycline (group D+) had the highest pH of cecal digesta (*P* < 0.05 vs. other groups except C+; see the significant A × Ch interaction). Four uninfected groups were characterized by the lowest cecal pH values (*P* < 0.05 vs. other groups).

**Table 1:** Response criteria of ileal and cecal digesta in turkeys on d 21 in Experiment 1.

	Ileum			Ceca		
	DM (%)	Viscosity (mPa⋅s)	pH	DM (%)	Ammonia (mg/g)	pH
Antibiotic (A)^1^						
C	18.5	2.29	6.58	12.9	0.255	4.96
M	16.8	2.23	7.03	12.5	0.239	4.96
E	18.5	2.23	6.75	12.7	0.234	4.94
D	17.9	1.95	6.56	13.3	0.261	5.17
Challenge (Ch)^2^						
-	18.9	2.08	6.53	12.6	0.218	4.67
+	17.0	2.27	6.93	13.1	0.277	5.34
Interaction (A × Ch)						
C-	20.5^a^	2.00^ab^	6.19^c^	11.5	0.214^c^	4.52^c^
C+	16.4^bc^	2.57^a^	6.96^ab^	14.3	0.297^ab^	5.39^ab^
M-	18.7^ab^	1.98^ab^	6.74abc	12.5	0.222^c^	4.72^c^
M+	15.0^c^	2.48^a^	7.33^a^	12.4	0.255abc	5.20^b^
E-	18.8^ab^	2.15^ab^	6.54^bc^	12.5	0.226^c^	4.69^c^
E+	18.3^ab^	2.31^ab^	6.96^ab^	12.8	0.242^bc^	5.18^b^
D-	17.4^bc^	2.19^ab^	6.65^bc^	14.0	0.209^c^	4.75^c^
D+	18.4^ab^	1.71^b^	6.48^bc^	12.7	0.314^a^	5.60^a^
*SEM*	*0.270*	*0.057*	*0.064*	*0.267*	*0.007*	*0.054*
*P-value*						
A	*0.012*	*0.074*	*0.008*	*0.674*	*0.178*	*0.027*
Ch	*0.000*	*0.060*	*0.000*	*0.410*	*0.000*	*0.000*
A × Ch interaction	*0.000*	*0.001*	*0.015*	*0.059*	*0.009*	*0.041*

1 C, untreated control; M, treated with monensin (90 mg per kg of feed, for 21 d); E, treated with enrofloxacin (10 mg per kg of BW, added to drinking water for 5 consecutive days after hatching); D, treated with doxycycline (at a dose of 50 mg per kg of BW, added to drinking water for 5 consecutive days after hatching).

2 On d 15, birds were challenged with avian pathogenic *E. coli* (+) or served as an uninfected control group with no challenge (-).

a-c Means within the same column with different superscripts differ significantly (*P* < 0.05). DM, dry matter; SEM, standard error of the mean.

The extracellular, intracellular and total activities of cecal bacterial α-glucosidase increased in response to the APEC challenge, compared with uninfected birds, irrespective of the antibiotic factor (*P* < 0.05; Table 2). The release rate of α-glucosidase decreased following the challenge, regardless of the antibiotic factor (*P* < 0.05). The A × Ch interaction indicated that the extracellular activity of cecal bacterial β-glucosidase was highest in C+ turkeys (*P* < 0.05 vs. other groups except M-) and lowest in D- birds (*P* < 0.05 vs. C+, M-, M+). An A × Ch interaction was also noted for the intracellular and total activities of β-glucosidase as well as its release rate (%). D+ birds were characterized by increased intracellular and total activities of β-glucosidase (*P* < 0.05 vs. other groups except C+). The release rate of cecal bacterial β-glucosidase was highest in M- birds and lowest in D+ turkeys (*P* < 0.05 vs. other groups except M+ and *P* < 0.05 vs. other groups except E+, respectively).

**Table 2:** Extracellular, intracellular, and total activity of bacterial α- and β-glucosidase in the cecal digesta in turkeys on d 21 in Experiment 1.

	α-Glucosidase (µmol/h/g)				β-Glucosidase (µmol/h/g)			
	Extracellular	Intracellular	Total	RR (%)	Extracellular	Intracellular	Total	RR (%)
Antibiotic (A)^1^								
C	7.58	9.04	16.6	49.1	1.63	2.60	4.23	40.8
M	6.18	11.2	17.4	38.9	1.76	1.06	2.82	63.2
E	8.36	10.6	19.0	48.6	0.68	1.97	2.65	31.7
D	8.37	13.0	21.4	44.3	0.66	3.70	4.36	20.5
Challenge (Ch)^2^								
-	5.45^b^	5.06^b^	10.5^b^	52.3^a^	1.06	1.47	2.52	42.1
+	9.79^a^	16.80^a^	26.6^a^	38.2^b^	1.31	3.19	4.50	36.0
Interaction (A × Ch)								
C-	5.61	4.07	9.68	58.0	0.93^cd^	1.97^b^	2.90^c^	32.5^d^
C+	9.55	14.0	23.6	40.2	2.34^a^	3.22^ab^	5.56^ab^	49.2^bc^
M-	4.03	6.10	10.1	40.1	2.13^ab^	1.05^b^	3.18^bc^	66.6^a^
M+	8.33	16.0	24.4	37.7	1.39^bc^	1.07^b^	2.46^c^	59.9^ab^
E-	6.35	4.63	11.0	57.5	0.71^cd^	0.95^b^	1.66^c^	42.1^cd^
E+	10.4	16.6	26.9	39.8	0.65^cd^	2.98^b^	3.63^bc^	21.4^de^
D-	5.83	5.43	11.3	53.6	0.45^d^	1.89^b^	2.35^c^	27.4^d^
D+	10.9	20.6	31.5	35.0	0.86^cd^	5.51^a^	6.37^a^	13.7^e^
*SEM*	*0.481*	*1.075*	*1.464*	*1.839*	*0.100*	*0.259*	*0.269*	*2.787*
*P-value*								
A	*0.190*	*0.371*	*0.413*	*0.084*	*0.000*	*0.000*	*0.003*	*0.000*
Ch	*0.000*	*0.000*	*0.000*	*0.000*	*0.035*	*0.000*	*0.000*	*0.099*
A × Ch	*0.957*	*0.615*	*0.713*	*0.201*	*0.000*	*0.017*	*0.001*	*0.004*

1 C, untreated control; M, treated with monensin (90 mg per kg of feed, for 21 d); E, treated with enrofloxacin (10 mg per kg of BW, added to drinking water for 5 consecutive days after hatching); D, treated with doxycycline (at a dose of 50 mg per kg of BW, added to drinking water for 5 consecutive days after hatching).

2 On d 15, birds were challenged with avian pathogenic *E. coli* (+) or served as an uninfected control group with no challenge (-).

a-e Means within the same column with different superscripts differ significantly (*P* < 0.05). RR, release rate; SEM, standard error of the mean.

Two-way ANOVA revealed that irrespective of the antibiotic factor, the APEC challenge enhanced the intracellular and total activities of cecal α-galactosidase and decreased its extracellular activity, relative to uninfected turkeys (*P* < 0.05; Table 3). Regardless of the Ch factor, the α-galactosidase extracellular activity decreased in response to the administration of monensin (**M**) and enrofloxacin (**E**) (*P* < 0.05 vs. C). Group C excelled all other groups with respect to the release rate of cecal α-galactosidase (*P* < 0.05; see A × Ch). The extracellular, intracellular and total activities of cecal β-galactosidase were highest in groups C+ and D+ (significant A × Ch). Regardless of the antibiotic factor, the Ch decreased the β-galactosidase release rate (*P* < 0.05 vs. unchallenged turkeys).

**Table 3:** Extracellular, intracellular, and total activity of bacterial α- and β-galactosidase in the cecal digesta in turkeys on d 21 in Experiment 1.

	α-Galactosidase (µmol/h/g)				β-Galactosidase (µmol/h/g)			
	Extracellular	Intracellular	Total	RR (%)	Extracellular	Intracellular	Total	RR (%)
Antibiotic (A)^1^								
C	8.23^a^	28.1	36.3	30.2	11.1	26.4	37.5	23.7
M	4.92^b^	40.1	45.0	12.3	6.49	16.8	23.3	26.3
E	4.61^b^	33.4	38.0	14.0	7.35	12.7	20.1	30.6
D	5.57^ab^	37.6	43.1	18.8	9.51	23.7	33.2	25.6
Challenge (Ch)^2^								
-	6.86^a^	19.8^b^	26.7^b^	28.2	1.49	7.07	8.57	19.4^b^
+	4.80^b^	49.8^a^	54.6^a^	9.49	15.7	32.8	48.5	33.7^a^
Interaction (A × Ch)								
C-	10.6	10.7	21.3	48.2^a^	1.24^c^	9.08^cd^	10.3^c^	14.9
C+	5.84	45.5	51.3	12.2^c^	20.9^a^	43.8^a^	64.6^a^	32.6
M-	5.15	31.3	36.4	15.4^c^	1.76^c^	8.40^cd^	10.2^c^	20.0
M+	4.69	48.9	53.6	9.28^c^	11.23^b^	25.3^b^	36.5^b^	32.5
E-	5.13	22.8	27.9	19.2^bc^	1.13^c^	4.53^d^	5.66^c^	20.8
E+	4.09	44.0	48.1	8.75^c^	13.56^b^	20.9^bc^	34.5^b^	40.4
D-	6.56	14.5	21.0	29.9^b^	1.83^c^	6.29^d^	8.12^c^	21.7
D+	4.57	60.7	65.3	7.75^c^	17.2^ab^	41.1^a^	58.2^a^	29.5
*SEM*	*0.475*	*3.007*	*3.048*	*1.919*	*1.057*	*2.100*	*3.036*	*1.451*
*P-value*								
A	*0.018*	*0.275*	*0.567*	*0.000*	*0.006*	*0.000*	*0.000*	*0.178*
Ch	*0.020*	*0.000*	*0.000*	*0.000*	*0.000*	*0.000*	*0.000*	*0.000*
A × Ch	*0.306*	*0.117*	*0.230*	*0.000*	*0.004*	*0.001*	*0.000*	*0.240*

1 C, untreated control; M, treated with monensin (90 mg per kg of feed, for 21 d); E, treated with enrofloxacin (10 mg per kg of BW, added to drinking water for 5 consecutive days after hatching); D, treated with doxycycline (at a dose of 50 mg per kg of BW, added to drinking water for 5 consecutive days after hatching).

2 On d 15, birds were challenged with avian pathogenic *E. coli* (+) or served as an uninfected control group with no challenge (-).

a-d Means within the same column with different superscripts differ significantly (*P* < 0.05). RR, release rate; SEM, standard error of the mean.

The extracellular activity of cecal β-glucuronidase increased significantly in birds infected with APEC, regardless of the antibiotic factor (*P* < 0.05; Table 4). Irrespective of the Ch factor, the administration of enrofloxacin (**E**) and doxycycline (**D**) decreased the extracellular activity of β-glucuronidase. As indicated by the A × Ch interaction, the intracellular and total activities of β-glucuronidase were highest in birds C+ (*P* < 0.05 vs. all groups except M+, E+, D+ and *P* < 0.05 vs. all other groups, respectively). Two-way ANOVA revealed that irrespective of the antibiotic factor, infection with APEC decreased the release rate of β-glucuronidase. Doxycycline caused a decrease in the extracellular activity of α-arabinopyranosidase, vs. C and M birds, irrespective of the APEC factor. At the same time, infection with APEC induced an increase in the activity of the above enzyme. The intracellular and total activities of α-arabinopyranosidase were highest in group C+ (*P* < 0.05 vs. all groups except M-; see A × Ch). Irrespective of the Ch factor, the release rate of α-arabinopyranosidase was enhanced in E and D birds (*P* < 0.05 vs. C, M and *P* < 0.05 vs. C, respectively). The APEC challenge caused an increase in the extracellular and intracellular activities of β-xylosidase, irrespective of the antibiotic factor (*P* < 0.05). The E and D antibiotics induced a decrease in the extracellular activity of β-xylosidase relative to groups C and M, regardless of the Ch factor. The A × Ch interaction showed enhanced total activity of β-xylosidase in groups M+ and D+ (*P* < 0.05 vs. all uninfected groups). Regardless of the antibiotic factor, the challenge with APEC caused a significant decrease in the release rate of cecal β-xylosidase relative to unchallenged birds.

**Table 4:** Extracellular, intracellular, and total activity of bacterial β-glucuronidase, α-arabinopyranosidase and β-xylosidase in the cecal digesta in turkeys on d 21 in Experiment 1.

	β-Glucuronidase (µmol/h/g)				α-Arabinopyranosidase (µmol/h/g)				β-Xylosidase (µmol/h/g)			
	Extra	Intra	Total	RR (%)	Extra	Intra	Total	RR (%)	Extra	Intra	Total	RR (%)
Antibiotic (A)^1^												
C	4.77^a^	6.17	10.9	48.8	1.36^a^	3.70	5.06	30.7^c^	4.22^a^	6.43	10.7	45.0
M	3.92^ab^	6.01	9.92	40.0	1.33^a^	2.62	3.95	35.6^bc^	4.48^a^	8.40	12.9	41.4
E	3.09^b^	4.03	7.12	47.4	1.10^ab^	0.903	2.00	56.1^a^	2.13^b^	6.89	9.02	34.1
D	2.78^b^	4.53	7.31	46.1	0.838^b^	1.13	1.97	48.1^ab^	2.44^b^	7.83	10.3	39.5
Challenge (Ch)^2^												
-	2.19^b^	2.51	4.70	50.2^a^	1.02^b^	1.79	2.82	42.9	2.74^b^	2.80^b^	5.54	54.3^a^
+	5.08^a^	7.86	12.9	40.9^b^	1.29^a^	2.38	3.67	42.4	3.89^a^	11.98^a^	15.9	25.7^b^
Interaction (A × Ch)												
C-	2.84	1.91^c^	4.75^cd^	58.1	1.08	2.56^bc^	3.63^bc^	30.8	3.28	2.91	6.19^cd^	54.6
C+	6.69	10.4^a^	17.1^a^	39.4	1.64	4.84^a^	6.48^a^	30.7	5.16	9.95	15.1^ab^	35.3
M-	2.67	4.96^bc^	7.63^bc^	36.1	1.41	3.24^ab^	4.66^ab^	29.8	4.45	4.95	9.41^bc^	54.5
M+	5.17	7.05^ab^	12.2^b^	43.9	1.26	1.99^bc^	3.24bcd	41.4	4.51	11.85	16.4^a^	28.3
E-	1.35	1.38^c^	2.73^d^	51.5	1.04	0.748^c^	1.79^cd^	58.9	1.67	2.31	3.98^cd^	48.7
E+	4.83	6.68^ab^	11.5^b^	43.4	1.15	1.06^bc^	2.21^cd^	53.4	2.59	11.47	14.1^ab^	19.6
D-	1.91	1.79^c^	3.71^cd^	55.3	0.557	0.626^c^	1.18^d^	52.0	1.58	1.02	2.60^d^	59.2
D+	3.65	7.26^ab^	10.9^b^	36.9	1.12	1.64^bc^	2.76bcd	44.2	3.31	14.64	18.0^a^	19.8
*SEM*	*0.270*	*0.501*	*0.685*	*2.094*	*0.069*	*0.238*	*0.269*	*2.325*	*0.241*	*0.757*	*0.862*	*2.542*
*P-value*												
A	*0.001*	*0.072*	*0.001*	*0.409*	*0.013*	*0.000*	*0.000*	*0.000*	*0.000*	*0.454*	*0.088*	*0.194*
Ch	*0.000*	*0.000*	*0.000*	*0.020*	*0.032*	*0.105*	*0.028*	*0.900*	*0.005*	*0.000*	*0.000*	*0.000*
A × Ch	*0.147*	*0.016*	*0.005*	*0.066*	*0.107*	*0.009*	*0.002*	*0.330*	*0.334*	*0.052*	*0.045*	*0.259*

1 C, untreated control; M, treated with monensin (90 mg per kg of feed, for 21 d); E, treated with enrofloxacin (10 mg per kg of BW, added to drinking water for 5 consecutive days after hatching); D, treated with doxycycline (at a dose of 50 mg per kg of BW, added to drinking water for 5 consecutive days after hatching).

2 On d 15, birds were challenged with avian pathogenic *E. coli* (+) or served as an uninfected control group with no challenge (-).

a-d Means within the same column with different superscripts differ significantly (*P* < 0.05). RR, release rate; SEM, standard error of the mean.

An A × Ch interaction was noted for the cecal concentrations of acetic acid (**C2**), butyric acid (**C4**), total SCFAs and putrefactive SCFAs (Table 5). The cecal concentrations of C2, C4, and total SCFAs were highest in group D+, whereas group D- was characterized by the lowest C2 content. C- turkeys had the lowest concentrations of C4 and PSCFAs (*P* < 0.05 vs. C+, M-, M+, D+ and *P* < 0.05 vs. M-, respectively). The concentrations of total SCFAs were lowest in groups C-, E+, and D- (*P* < 0.05 vs. D+). Regardless of the antibiotic factor, the challenge induced an increase in the cecal concentrations of propionic acid (**C3**) and iso-valeric acid (*P* < 0.05). Group C- was characterized by the highest proportion of C2 and the lowest proportion of C4 (% of total SCFAs). The opposite situation was observed in group D+ (see A × Ch). The proportion of C3 was highest in E turkeys, regardless of Ch (*P* < 0.05 vs. M and D). Irrespective of the antibiotic factor, APEC infection increased the percentage of C3 relative to total SCFAs (*P* < 0.05).

**Table 5:** Concentrations and profile of short-chain fatty acids in the cecal digesta in turkeys on d 21 in Experiment 1.

	SCFAs (µmol/g digesta)								SCFA profile (% of total SCFAs)		
	C2	C3	C4i	C4	C5i	C5	PSCFA	Total	C2	C3	C4
Antibiotic (A)^1^											
C	43.1	2.23	0.115	6.73	0.090	0.042	0.247	52.3	83.0	4.34^ab^	12.3
M	44.2	1.99	0.396	8.35	0.132	0.187	0.715	55.2	79.4	3.79^b^	15.6
E	42.5	2.67	0.181	6.28	0.083	0.000	0.264	51.7	81.6	5.39^a^	12.5
D	42.3	2.09	0.201	10.4	0.105	0.090	0.395	55.2	77.2	3.71^b^	18.5
Challenge (Ch)^2^											
-	43.5	1.79^b^	0.218	6.25	0.046^b^	0.058	0.322	51.8	83.6	3.58^b^	12.2
+	42.6	2.70^a^	0.228	9.62	0.159^a^	0.101	0.489	55.4	76.9	5.03^a^	17.2
Interaction (A × Ch)											
C-	41.4abc	1.79	0.016	3.14^c^	0.000	0.000	0.016^b^	46.3^b^	89.0^a^	4.10	6.89^c^
C+	44.8abc	2.67	0.215	10.3^b^	0.180	0.084	0.479^ab^	58.2^ab^	77.0^bc^	4.57	17.7^ab^
M-	49.7^ab^	1.52	0.582	9.45^bd^	0.083	0.233	0.898^a^	61.5^ab^	79.9^bc^	2.54	16.0^ab^
M+	38.7abc	2.46	0.210	7.26^bd^	0.181	0.141	0.532^ab^	48.9^ab^	78.9^bc^	5.04	15.1^ab^
E-	48.6^ab^	2.30	0.188	6.18^cd^	0.064	0.000	0.252^ab^	57.3^ab^	84.7^ab^	4.07	10.8^bc^
E+	36.4^bc^	3.04	0.174	6.39^cd^	0.101	0.000	0.275^ab^	46.1^b^	78.5^bc^	6.71	14.2^bc^
D-	34.2^c^	1.56	0.086	6.25^cd^	0.035	0.000	0.121^ab^	42.1^b^	80.9^bc^	3.60	15.2^ab^
D+	50.5^a^	2.62	0.315	14.5^a^	0.175	0.179	0.670^ab^	68.3^a^	73.5^c^	3.82	21.8^a^
*SEM*	*1.576*	*0.134*	*0.054*	*0.498*	*0.013*	*0.027*	*0.069*	*1.875*	*0.811*	*0.246*	*0.764*
*P-value*											
A	*0.971*	*0.230*	*0.291*	*0.000*	*0.407*	*0.085*	*0.042*	*0.807*	*0.010*	*0.026*	*0.001*
Ch	*0.766*	*0.001*	*0.923*	*0.000*	*0.000*	*0.416*	*0.193*	*0.281*	*0.000*	*0.001*	*0.000*
A × Ch	*0.003*	*0.974*	*0.182*	*0.000*	*0.129*	*0.315*	*0.049*	*0.000*	*0.027*	*0.086*	*0.008*

1 C, untreated control; M, treated with monensin (90 mg per kg of feed, for 21 d); E, treated with enrofloxacin (10 mg per kg of BW, added to drinking water for 5 consecutive days after hatching); D, treated with doxycycline (at a dose of 50 mg per kg of BW, added to drinking water for 5 consecutive days after hatching).

2 On d 15, birds were challenged with avian pathogenic *E. coli* (+) or served as an uninfected control group with no challenge (-).

a-d Means within the same column with different superscripts differ significantly (*P* < 0.05). SEM, standard error of the mean. C2, acetic acid; C3, propionic acid; C4i, iso-butyric acid; C4, butyric acid; C5i, iso-valeric acid; C5, valeric acid; PSCFA, putrefactive short-chain fatty acids; SCFA, short-chain fatty acids.

### Experiment 2

Irrespective of the Ch factor, ileal DM concentration decreased significantly in response to enrofloxacin, *versus* C, D, M (Table 6). Early APEC infection (R; on d 15) increased ileal DM percentage (*P* < 0.05 vs. unchallenged N birds; regardless of the antibiotic factor). The A × Ch interaction showed increased ileal viscosity in CR birds, compared with unchallenged CN turkeys (*P* < 0.05). Antibiotic M, E, D factors in early-infected birds (MR, ER, DR) lowered ileal viscosity to insignificant values relative to unchallenged CN turkeys. Regardless of the Ch factor, enrofloxacin (E) lowered ileal pH vs. monensin (*P* < 0.05). Irrespective of the antibiotic factor, both early (R) and late (L) challenges increased the pH of ileal digesta vs. N turkeys (*P* < 0.05). An A × Ch interaction revealed that CR birds, but not CL ones, had decreased cecal DM concentration vs. CN turkeys. Cecal ammonia concentration was significantly decreased by the administration of M, E, D in turkeys subjected to L infection as compared with group CL (see A × Ch).

**Table 6:** Response criteria of ileal and cecal digesta in turkeys on d 56 in Experiment 2.

	Ileum			Ceca		
	DM (%)	Viscosity (mPa⋅s)	pH	DM (%)	Ammonia (mg/g)	pH
Antibiotic (A)^1^						
C	18.79^a^	2.07	6.26^ab^	13.34	0.294	5.85
M	18.63^a^	1.93	6.55^a^	14.44	0.246	5.75
E	17.13^b^	1.80	6.10^b^	15.18	0.268	6.14
D	18.96^a^	1.94	6.51^ab^	14.58	0.268	6.13
*E.coli* challenge (Ch)^2^						
N	18.02^b^	1.67	5.83^b^	14.97	0.251	5.87
R	18.96^a^	2.20	6.64^a^	14.89	0.284	6.13
L	18.18^ab^	1.94	6.59^a^	13.29	0.272	5.90
Interaction (A × Ch)						
CN	18.54	1.66^bc^	5.43	15.74^ab^	0.235^cd^	5.78abc
CR	19.73	2.52^a^	6.65	11.14^c^	0.318^ab^	5.46^c^
CL	18.10	2.04abc	6.69	13.14^bc^	0.330^a^	6.33^ab^
MN	17.80	1.54^c^	5.94	13.99abc	0.219^d^	5.65^bc^
MR	18.81	2.22^ab^	7.01	15.16^ab^	0.263^bd^	5.98abc
ML	19.27	2.05abc	6.71	14.18^bc^	0.255^bd^	5.63^bc^
EN	16.46	1.52^c^	5.56	15.01^ab^	0.279abcd	6.05abc
ER	18.36	1.97abc	6.37	17.29^a^	0.265abcd	6.46^ab^
EL	16.50	1.90abc	6.38	13.23^bc^	0.259bcd	5.88abc
DN	19.28	1.97abc	6.39	15.14^ab^	0.272abcd	6.00abc
DR	18.96	2.06abc	6.54	15.97^ab^	0.289abc	6.62^a^
DL	18.63	1.79^bc^	6.59	12.63^bc^	0.244^cd^	5.75^bc^
*SEM*	*0.177*	*0.046*	*0.073*	*0.264*	*0.005*	*0.061*
*P-value*						
A	*0.000*	*0.096*	*0.028*	*0.033*	*0.001*	*0.024*
Ch	*0.028*	*0.000*	*0.000*	*0.003*	*0.005*	*0.096*
A × Ch	*0.125*	*0.046*	*0.125*	*0.000*	*0.000*	*0.001*

1 C, untreated control; M, treated with monensin (90 mg per kg of feed, for 56 d); E, treated with enrofloxacin (10 mg per kg of BW, added to drinking water for 5 consecutive days after hatching); D, treated with doxycycline (at a dose of 50 mg per kg of BW, added to drinking water for 5 consecutive days after hatching).

2 On d 15 (R) or d 50 (L), birds were challenged with avian pathogenic *E. coli* or served as a control group with no challenge (N).

a-c Means within the same column with different superscripts differ significantly (*P* < 0.05). DM, dry matter; SEM, standard error of the mean.

Irrespective of the Ch factor, the administration of E and D decreased the extracellular and total activities of cecal bacterial α-glucosidase (*P* < 0.05 vs. C, M; Table 7). Regardless of the antibiotic factor, the early challenge, but not the late one, increased the intracellular and total activities of cecal α-glucosidase vs. N turkeys. An A × Ch interaction showed the highest release rate of α-glucosidase in EN birds (*P* < 0.05 vs. all M groups, ER, EL, DL). The highest extracellular activity of β-glucosidase was noted in groups CR, CL, MR, and EL (*P* < 0.05 vs. CN, DN, DR). The release rate of β-glucosidase was highest in groups EN and DN (see A × Ch).

**Table 7:** Cecal activity of bacterial α- and β-glucosidase in turkeys on d 56 in Experiment 2.

	α-Glucosidase (µmol/h/g digesta)				β-Glucosidase (µmol/h/g digesta)			
	Extracellular	Intracellular	Total	RR (%)	Extracellular	Intracellular	Total	RR (%)
Antibiotic (A)^1^								
C	19.26^a^	15.37^b^	34.62^a^	57.90	3.60	5.49	9.09	42.29
M	19.70^a^	22.33^a^	42.02^a^	47.70	3.49	10.03	13.52	27.43
E	11.24^b^	8.31^c^	19.55^b^	59.22	3.82	5.12	8.94	49.52
D	14.79^b^	10.58^bc^	25.37^b^	60.65	2.10	4.30	6.40	45.26
Challenge (Ch)^2^								
N	16.08	11.57^b^	27.65^b^	62.16	2.49	4.90	7.39	48.38
R	17.38	16.99^a^	34.37^a^	52.52	3.30	6.26	9.56	35.46
L	15.28	13.87^ab^	29.14^ab^	54.42	3.97	7.54	11.51	39.54
Interaction (A × Ch)								
CN	20.85	17.41	38.26	54.05abc	1.75^b^	7.49^bc^	9.24^bc^	18.25^f^
CR	17.22	13.51	30.73	57.86abc	4.71^a^	4.88^c^	9.58^bc^	51.29bcd
CL	19.70	15.18	34.88	61.78abc	4.35^a^	4.10^c^	8.45^bc^	57.34abc
MN	19.33	19.19	38.52	50.81^c^	2.66^ab^	10.61abc	13.27^ab^	20.47^f^
MR	20.99	29.10	50.09	43.02^c^	4.32^a^	6.72^bc^	11.04^ab^	38.88cdef
ML	18.77	18.68	37.46	49.27^c^	3.48^ab^	12.77^a^	16.25^a^	22.95^ef^
EN	12.21	4.62	16.83	73.35^a^	3.79^ab^	1.08^de^	4.87^cd^	76.16^a^
ER	12.50	11.00	23.50	51.30^bc^	2.69^ab^	6.78^c^	9.47^bc^	30.16def
EL	9.00	9.31	18.31	53.01^bc^	4.99^a^	7.50^c^	12.49^ab^	42.23bcde
DN	11.93	5.08	17.00	70.42^ab^	1.76^b^	0.43^e^	2.19^d^	78.65^a^
DR	18.80	14.37	33.17	57.88abc	1.49^b^	6.69^c^	8.17^bc^	21.49^ef^
DL	13.64	12.29	25.93	53.63^bc^	3.06^ab^	5.79bcd	8.84^bc^	35.65cdef
*SEM*	*0.676*	*0.959*	*1.461*	*1.395*	*0.180*	*0.440*	*0.484*	*2.402*
*P-value*								
A	*0.000*	*0.000*	*0.000*	*0.001*	*0.000*	*0.000*	*0.000*	*0.000*
Ch	*0.327*	*0.014*	*0.041*	*0.003*	*0.000*	*0.002*	*0.000*	*0.000*
A × Ch	*0.218*	*0.052*	*0.071*	*0.008*	*0.001*	*0.000*	*0.001*	*0.000*

1 C, untreated control; M, treated with monensin (90 mg per kg of feed, for 56 d); E, treated with enrofloxacin (10 mg per kg of BW, added to drinking water for 5 consecutive days after hatching); D, treated with doxycycline (at a dose of 50 mg per kg of BW, added to drinking water for 5 consecutive days after hatching).

2 On d 15 (R) or d 50 (L), birds were challenged with avian pathogenic *E. coli* or served as a control group with no challenge (N).

a-f Means within the same column with different superscripts differ significantly (*P* < 0.05). RR, release rate; SEM, standard error of the mean.

A significant A × Ch indicated that group CL excelled all remaining groups with respect to the extracellular activity of cecal bacterial α-galactosidase (Table 8). Groups EN and DN were characterized by extremely low intracellular and total activities of this enzyme (see A × Ch). An A × Ch interaction indicated that the release rate of cecal α-galactosidase was highest in group EN, and lowest in groups ER and DR subjected to the early challenge. Group DR was characterized by the highest increase in the extracellular, intracellular and total activities of cecal bacterial β-galactosidase (see A × Ch). Groups EN and DN had the highest release rate of cecal β-galactosidase, whereas the lowest values were observed in groups MR, ER, and DR (A × Ch interaction).

**Table 8:** Cecal activity of bacterial α- and β-galactosidase in turkeys on d 56 in Experiment 2.

	α-Galactosidase (µmol/h/g digesta)				β-Galactosidase (µmol/h/g digesta)			
	Extracellular	Intracellular	Total	RR (%)	Extracellular	Intracellular	Total	RR (%)
Antibiotic (A)^1^								
C	27.88	77.38	105.26	27.14	30.81	61.19	92.00	34.61
M	19.76	79.56	99.31	21.78	23.86	47.04	70.90	32.96
E	14.84	40.27	55.11	38.71	14.40	41.51	55.91	38.07
D	14.23	57.16	71.39	30.59	24.61	65.26	89.86	35.81
Challenge (Ch)^2^								
N	15.92	40.03	55.96	42.21	23.23	31.35	54.57	46.62
R	14.09	91.65	105.74	13.49	23.51	83.49	107.00	24.36
L	27.52	59.09	86.61	32.97	23.51	46.41	69.93	35.11
Interaction (A × Ch)								
CN	14.60^bc^	83.62^ab^	98.22^ab^	15.31^ef^	32.63abc	54.30^bc^	86.93^bc^	37.30^bc^
CR	20.34^bc^	83.99^ab^	104.32^a^	19.91^ef^	25.79abcd	49.58bcd	75.37bcd	34.09bcd
CL	48.71^a^	64.53abc	113.24^a^	46.19^bc^	34.01^ab^	79.69^b^	113.70^b^	32.43bcd
MN	17.13^bc^	57.00^bc^	74.13abc	27.60^de^	22.58bcd	46.59bcd	69.18bcd	32.46bcd
MR	19.26^bc^	99.66^ab^	118.92^a^	16.52^ef^	17.18bcd	48.89bcd	66.06bcd	26.18^cd^
ML	22.88^b^	82.01^ab^	104.90^a^	21.21^ef^	31.81abc	45.64bcd	77.45bcd	40.25^bc^
EN	16.72^bc^	8.40^d^	25.11^c^	66.17^a^	21.54bcd	11.44^d^	32.98^d^	65.58^a^
ER	6.05^c^	80.75^ab^	86.81^ab^	7.91^f^	10.38^d^	86.89^b^	97.28^bc^	15.00^d^
EL	21.75^bc^	31.66^cd^	53.40^bc^	42.06^cd^	11.27^d^	26.20^cd^	37.47^d^	33.63bcd
DN	15.25^bc^	11.11^d^	26.37^c^	59.75^ab^	16.16^cd^	13.04^cd^	29.20^d^	51.14^ab^
DR	10.70^bc^	102.21^a^	112.91^a^	9.60^f^	40.69^a^	148.62^a^	189.30^a^	22.18^cd^
DL	16.74^bc^	58.16^bc^	74.90abc	22.43^ef^	16.97^cd^	34.12^cd^	51.09^cd^	34.12bcd
*SEM*	*1.375*	*3.999*	*4.335*	*2.097*	*1.353*	*4.412*	*5.139*	*1.723*
*P-value*								
A	*0.000*	*0.000*	*0.000*	*0.000*	*0.000*	*0.004*	*0.000*	*0.510*
Ch	*0.000*	*0.000*	*0.000*	*0.000*	*0.991*	*0.000*	*0.000*	*0.000*
A × Ch	*0.000*	*0.000*	*0.022*	*0.000*	*0.000*	*0.000*	*0.000*	*0.000*

1 C, untreated control; M, treated with monensin (90 mg per kg of feed, for 56 d); E, treated with enrofloxacin (10 mg per kg of BW, added to drinking water for 5 consecutive days after hatching); D, treated with doxycycline (at a dose of 50 mg per kg of BW, added to drinking water for 5 consecutive days after hatching).

2 On d 15 (R) or d 50 (L), birds were challenged with avian pathogenic *E. coli* or served as a control group with no challenge (N).

a-f Means within the same column with different superscripts differ significantly (*P* < 0.05). RR, release rate; SEM, standard error of the mean.

The A × Ch interaction indicated that CL turkeys had the highest extracellular activity of cecal bacterial β-glucuronidase (*P* < 0.05 vs. all other groups), while the lowest activity of this enzyme was noted in turkeys from groups MR, EN, ER, and DN (Table 9). The total activity of β-glucuronidase was most enhanced in both challenged (early and late) groups C (CR, CL). Groups CL and ER were characterized by the highest and lowest release rates of β-glucuronidase, respectively (see the A × Ch interaction). An A × Ch interaction revealed that the extracellular, intracellular and total activities of cecal bacterial α-arabinopyranosidase were highest in ER and DR turkeys. Regardless of the A factor, the late challenge induced an increase in the release rate of cecal α-arabinopyranosidase in comparison with early-challenged turkeys (*P* < 0.05). An A × Ch showed the highest extracellular activity of cecal β-xylosidase in groups CL, MR, and EL (Table 10). The total activity of this enzyme was enhanced in group MR (*P* < 0.05 vs. all remaining groups; A × Ch). In turkeys administered enrofloxacin (E), R and L challenges caused a significant decrease in the release rate of cecal β-xylosidase, compared with uninfected EN birds (see the A × Ch interaction).

**Table 9:** Cecal activity of bacterial β-glucuronidase and α-arabinopyranosidase in turkeys on d 56 in Experiment 2.

	β-Glucuronidase (µmol/h/g digesta)				α-Arabinopyranosidase (µmol/h/g digesta)			
	Extracellular	Intracellular	Total	RR (%)	Extracellular	Intracellular	Total	RR (%)
Antibiotic (A)^1^								
C	25.68	36.75	62.42	40.19	2.01	4.55	6.57	35.02
M	11.41	27.86^b^	39.27	31.85	1.82	3.79	5.61	34.69
E	8.59	16.04	24.63	37.22	2.60	5.44	8.04	39.04
D	10.70	22.11	32.81	36.74	2.37	6.16	8.53	33.63
Challenge (Ch)^2^								
N	9.04	20.52	29.56	38.33	1.63	3.73	5.36	35.52^ab^
R	12.07	29.31	41.38	28.59	2.53	7.62	10.15	29.40^b^
L	21.18	27.24	48.41	42.59	2.45	3.60	6.05	41.87^a^
Interaction (A × Ch)								
CN	12.76^bc^	32.11abc	44.87abc	29.82^bc^	1.58^bc^	4.81abc	6.40^b^	26.25
CR	23.90^b^	45.18^a^	69.08^a^	36.43abc	1.35^c^	5.25abc	6.60^b^	27.78
CL	40.37^a^	32.95abc	73.32^a^	54.32^a^	3.11^ab^	3.59^bc^	6.71^b^	51.02
MN	11.45^bc^	31.94abc	43.39abc	36.83abc	1.58^bc^	3.26^c^	4.84^b^	37.98
MR	4.88^c^	14.40^bc^	19.28^cd^	27.79^bc^	1.45^bc^	4.13^bc^	5.58^b^	28.06
ML	17.90^bc^	37.24^ab^	55.15^ab^	30.93^bc^	2.43abc	3.97^bc^	6.40^b^	38.02
EN	6.51^c^	7.45^c^	13.97^c^	48.23^ab^	2.06abc	3.04^c^	5.10^b^	43.70
ER	5.10^c^	21.92abc	27.02^bc^	19.41^c^	3.66^a^	10.05^ab^	13.71^a^	31.89
EL	14.16^bc^	18.75^bc^	32.91^bc^	44.04^ab^	2.08abc	3.23^c^	5.31^b^	41.53
DN	5.45^c^	10.58^cd^	16.03^cd^	38.43abc	1.29^c^	3.79^bc^	5.08^b^	34.15
DR	14.37^bc^	35.75^ab^	50.13^ab^	30.73^bc^	3.66^a^	11.06^a^	14.72^a^	29.85
DL	12.27^bc^	20.00^bc^	32.27^bc^	41.05abc	2.17abc	3.62^bc^	5.79^b^	36.90
*SEM*	*1.255*	*1.840*	*2.604*	*1.612*	*0.129*	*0.456*	*0.516*	*1.650*
*P-value*								
A	*0.000*	*0.000*	*0.000*	*0.209*	*0.043*	*0.171*	*0.063*	*0.638*
Ch	*0.000*	*0.053*	*0.000*	*0.000*	*0.001*	*0.000*	*0.000*	*0.007*
A × Ch	*0.000*	*0.001*	*0.000*	*0.004*	*0.000*	*0.028*	*0.001*	*0.236*

1 C, untreated control; M, treated with monensin (90 mg per kg of feed, for 56 d); E, treated with enrofloxacin (10 mg per kg of BW, added to drinking water for 5 consecutive days after hatching); D, treated with doxycycline (at a dose of 50 mg per kg of BW, added to drinking water for 5 consecutive days after hatching).

2 On d 15 (R) or d 50 (L), birds were challenged with avian pathogenic *E. coli* or served as a control group with no challenge (N).

a-d Means within the same column with different superscripts differ significantly (*P* < 0.05). RR, release rate; SEM, standard error of the mean.

**Table 10:** Cecal activity of bacterial β-xylosidase in turkeys on d 56 in Experiment 2.

	β-Xylosidase (µmol/h/g digesta)			
	Extracellular	Intracellular	Total	RR (%)
Antibiotic (A)^1^				
C	2.94	19.48	22.42	15.53
M	3.21	41.38	44.60	8.30
E	3.13	14.47	17.60	28.17
D	2.84	17.46	20.29	18.04
Challenge (Ch)^2^				
N	2.71	14.16	16.87	25.60
R	2.57	35.48	38.05	7.81
L	3.80	19.96	23.76	19.13
Interaction (A × Ch)				
CN	2.57^ab^	21.40bcde	23.96^bc^	15.17cde
CR	1.44^b^	19.84bcde	21.28^bc^	8.93^de^
CL	4.81^a^	17.21bcde	22.02^bc^	22.47bcd
MN	2.08^ab^	24.43bcde	26.51^bc^	10.50^de^
MR	4.45^a^	65.47^a^	69.91^a^	6.50^e^
ML	3.11^ab^	34.25^b^	37.36^b^	7.90^de^
EN	3.47^ab^	3.81^e^	7.28^c^	47.95^a^
ER	1.35^b^	27.87bcd	29.22^bc^	5.08^e^
EL	4.57^a^	11.73^ce^	16.30^bc^	31.49^b^
DN	2.73^ab^	6.99^de^	9.73^c^	28.78^bc^
DR	3.05^ab^	28.75^bc^	31.80^b^	10.71^de^
DL	2.73^ab^	16.63bcde	19.35^bc^	14.65cde
*SEM*	*0.200*	*1.981*	*2.030*	*1.521*
*P-value*				
A	*0.871*	*0.000*	*0.000*	*0.000*
Ch	*0.010*	*0.000*	*0.000*	*0.000*
A × Ch	*0.000*	*0.001*	*0.001*	*0.000*

1 C, untreated control; M, treated with monensin (90 mg per kg of feed, for 56 d); E, treated with enrofloxacin (10 mg per kg of BW, added to drinking water for 5 consecutive days after hatching); D, treated with doxycycline (at a dose of 50 mg per kg of BW, added to drinking water for 5 consecutive days after hatching).

2 On d 15 (R) or d 50 (L), birds were challenged with avian pathogenic *E. coli* or served as a control group with no challenge (N).

a-e Means within the same column with different superscripts differ significantly (*P* < 0.05). RR, release rate; SEM, standard error of the mean.

An A × Ch interaction was noted for the cecal concentrations of all SCFAs except iso-valeric acid (Table 11). The concentrations of individual acids and total SCFAs were lowest in EN and DN turkeys. Regardless of the Ch factor, the cecal concentration of iso-valeric acid was lower in birds treated with enrofloxacin (E) and doxycycline (D) vs. M (*P* < 0.05). A significant A × Ch interaction was also noted for the cecal SCFA profile. In enrofloxacin-treated birds, both early and late challenges induced a significant decrease in the proportion of C2 and an increase in the proportion of C4 (% of total SCFAs, *P* < 0.05 vs. EN). In the case of doxycycline, the early challenge (DR) decreased the proportion of C2 and increased the proportion of C4 vs. group DN. Regardless of the A factor, both APEC challenges (early and late) led to a significant decrease in the cecal concentrations of ω-muricholic acid and deoxycholic acid (*P* < 0.05 vs. uninfected turkeys; Table 12). In addition, the R challenge reduced the levels of β-muricholic acid vs. uninfected birds (*P* < 0.05). An A × Ch interaction pointed to a significant increase in the cecal concentration of lithocholic acid in MR and DR birds, compared with their counterparts (MR vs. MN and ML, DR vs. DN).

**Table 11:** Cecal SCFA concentrations and profile in turkeys on d 56 in Experiment 2.

	SCFA concentrations (µmol/g digesta)								SCFA profile (% of total SCFAs)		
	C2	C3	C4i	C4	C5i	C5	PSCFA	Total	C2	C3	C4
Antibiotic (A)^1^											
C	64.49	7.30	0.530	15.09	0.491^ab^	0.552	1.574	88.47	72.48	8.44	17.25
M	65.15	7.92	0.665	16.90	0.789^a^	0.722	2.166	92.15	70.36	8.97	18.21
E	41.57	4.50	0.343	14.70	0.321^b^	0.386	1.050	61.82	71.92	7.15	19.57
D	42.89	3.29	0.337	10.10	0.333^b^	0.236	0.906	57.18	79.05	4.63	15.09
Challenge (Ch)^2^											
N	38.99	5.05	0.335	7.15	0.336	0.308	0.979	52.17^b^	79.36	7.50	11.92
R	60.01	5.32	0.452	19.53	0.505	0.552	1.508	86.38^a^	69.23	6.34	22.61
L	61.58	6.88	0.619	15.92	0.610	0.562	1.783	86.16^a^	71.77	8.04	18.05
Interaction (A × Ch)											
CN	71.50^a^	8.48^ab^	0.629^ab^	13.23^b^	0.535	0.636^a^	1.799^ab^	95.02^a^	75.29bcd	9.22^ab^	13.62bcd
CR	66.03^a^	5.92abc	0.286^ab^	15.77^ab^	0.163	0.373^ab^	0.822^ab^	88.55^a^	74.51bcd	6.69abc	17.88bcd
CL	55.95^a^	7.51^ab^	0.676^ab^	16.28^ab^	0.777	0.647^a^	2.100^a^	81.83^a^	67.64^cd^	9.43^ab^	20.25abc
MN	71.34^a^	10.75^a^	0.712^a^	13.76^b^	0.736	0.597^ab^	2.045^a^	97.90^a^	71.86bcd	11.80^a^	14.15bcd
MR	55.30^a^	7.42^ab^	0.548^ab^	19.47^ab^	0.961	0.873^a^	2.382^a^	84.58^a^	65.43^d^	9.01^ab^	22.62^ab^
ML	68.80^a^	5.58abcd	0.734^a^	17.47^ab^	0.671	0.696^a^	2.070^a^	93.96^a^	73.80bcd	6.09abc	17.84bcd
EN	9.64^b^	0.88^cd^	0.000^b^	1.070^c^	0.061	0.000^b^	0.061^b^	11.65^b^	82.83^ab^	7.39abc	9.27^d^
ER	54.81^a^	3.79bcd	0.424^ab^	24.30^a^	0.347	0.588^ab^	1.359^ab^	84.26^a^	64.50^d^	4.74^bc^	29.17^a^
EL	60.26^a^	8.83^ab^	0.605^ab^	18.73^ab^	0.554	0.570^ab^	1.729^ab^	89.56^a^	68.42^cd^	9.31^ab^	20.27abc
DN	3.48^b^	0.09^d^	0.000^b^	0.522^c^	0.012	0.000^b^	0.012^b^	4.10^b^	87.46^a^	1.61^c^	10.64^cd^
DR	63.90^a^	4.16bcd	0.550^ab^	18.59^ab^	0.548	0.373^ab^	1.471^ab^	88.12^a^	72.47bcd	4.93^bc^	20.77^ab^
DL	61.29^a^	5.61abcd	0.460^ab^	11.18^b^	0.439	0.335^ab^	1.235^ab^	79.31^a^	77.21abc	7.34abc	13.86bcd
*SEM*	*2.567*	*0.454*	*0.048*	*0.879*	*0.059*	*0.044*	*0.134*	*3.507*	*0.928*	*0.475*	*0.781*
*P-value*											
A	*0.000*	*0.000*	*0.022*	*0.000*	*0.011*	*0.000*	*0.001*	*0.000*	*0.000*	*0.002*	*0.062*
Ch	*0.000*	*0.078*	*0.031*	*0.000*	*0.127*	*0.010*	*0.021*	*0.000*	*0.000*	*0.240*	*0.000*
A × Ch	*0.000*	*0.000*	*0.038*	*0.000*	*0.132*	*0.048*	*0.033*	*0.000*	*0.000*	*0.005*	*0.007*

1 C, untreated control; M, treated with monensin (90 mg per kg of feed, for 56 d); E, treated with enrofloxacin (10 mg per kg of BW, added to drinking water for 5 consecutive days after hatching); D, treated with doxycycline (at a dose of 50 mg per kg of BW, added to drinking water for 5 consecutive days after hatching).

2 On d 15 (R) or d 50 (L), birds were challenged with avian pathogenic *E. coli* or served as a control group with no challenge (N).

a-d Means within the same column with different superscripts differ significantly (*P* < 0.05). C2, acetic acid; C3, propionic acid; C4i, iso-butyric acid; C4, butyric acid; C5i, iso-valeric acid; C5, valeric acid; PSCFA, putrefactive short-chain fatty acids; SCFA, short-chain fatty acids; SEM, standard error of the mean.

**Table 12:** Cecal bile acid concentrations in turkeys on d 56 in Experiment 2.

	αMCA	βMCA	ωMCA	CA	HDCA	UDCA	CDCA	DCA	LCA	Total BA
Antibiotic (A)^1^										
C	1.000	1.125	0.637	3.136	0.184	0.144	1.020	2.249	17.084	26.578
M	1.127	0.709	0.673	4.198	0.108	0.204	1.294	1.758	15.983	26.052
E	0.457	0.641	0.614	3.201	0.183	0.129	0.723	1.148	16.237	23.332
D	1.287	0.468	0.564	3.985	0.111	0.078	1.329	1.363	17.489	26.672
Challenge (Ch)^2^										
N	1.116	1.167^a^	1.169^a^	3.988	0.210	0.173	1.392	3.998^a^	15.450	28.664
R	0.619	0.331^b^	0.227^b^	2.970	0.089	0.064	0.753	0.208^b^	18.485	23.746
L	1.168	0.708^ab^	0.470^b^	3.931	0.140	0.179	1.129	0.682^b^	16.159	24.566
Interaction (A × Ch)										
CN	0.743	2.370	1.346	3.524	0.394	0.116	1.145	5.571	18.352abc	33.561
CR	1.217	0.167	0.259	2.617	0.054	0.062	1.136	0.609	15.568abc	21.689
CL	1.041	0.838	0.306	3.266	0.105	0.253	0.780	0.566	17.331abc	24.486
MN	0.722	1.033	1.359	3.933	0.154	0.418	1.243	4.431	14.633^c^	27.925
MR	0.656	0.351	0.264	3.682	0.090	0.049	0.896	0.084	20.627^ab^	26.699
ML	2.003	0.743	0.396	4.978	0.081	0.146	1.741	0.758	12.688^c^	23.533
EN	0.092	0.516	0.636	2.614	0.128	0.064	0.632	2.724	14.201^c^	21.606
ER	0.420	0.266	0.180	2.676	0.098	0.084	0.596	0.075	16.851abc	21.246
EL	0.859	1.140	1.026	4.313	0.323	0.240	0.940	0.646	17.658abc	27.144
DN	2.906	0.750	1.335	5.880	0.167	0.096	2.547	3.268	14.615^bc^	31.564
DR	0.184	0.542	0.204	2.906	0.114	0.060	0.384	0.064	20.893^a^	25.352
DL	0.771	0.111	0.152	3.168	0.053	0.077	1.056	0.758	16.958abc	23.101
*SEM*	*0.229*	*0.125*	*0.108*	*0.336*	*0.031*	*0.037*	*0.140*	*0.301*	*0.555*	*1.034*
*P-value*										
A	*0.608*	*0.243*	*0.985*	*0.599*	*0.696*	*0.699*	*0.365*	*0.437*	*0.720*	*0.620*
Ch	*0.562*	*0.017*	*0.001*	*0.396*	*0.274*	*0.377*	*0.163*	*0.000*	*0.055*	*0.115*
A × Ch	*0.267*	*0.071*	*0.379*	*0.634*	*0.350*	*0.604*	*0.155*	*0.697*	*0.048*	*0.300*

1 C, untreated control; M, treated with monensin (90 mg per kg of feed, for 56 d); E, treated with enrofloxacin (10 mg per kg of BW, added to drinking water for 5 consecutive days after hatching); D, treated with doxycycline (at a dose of 50 mg per kg of BW, added to drinking water for 5 consecutive days after hatching).

2 On d 15 (R) or d 50 (L), birds were challenged with avian pathogenic *E. coli* or served as a control group with no challenge (N).

a-c Means within the same column with different superscripts differ significantly (*P* < 0.05). Bile acid (BA, µg/g): MCA, muricholic acid; CA, cholic acid; HDCA, hyodeoxycholic acid; UDCA, ursodeoxycholic acid; CDCA, chenodeoxycholic acid; DCA, deoxycholic acid; LCA, lithocholic acid; SEM, standard error of the mean.

## DISCUSSION

In the present study, turkeys were experimentally infected with APEC, which is a prevalent risk factor in poultry production (Huff et al., 2013). Turkeys were infected on d 15 or d 50 to mimic the stressor's occurrence in an early or later stage of rearing. To some extent, the fact that the infection had no effect on the body weight gain of turkeys was unexpected. However, such a possibility could be considered since the induced APEC infection was subclinical. In an experiment on broilers (Istiqomah et al., 2013), even a higher dose of APEC administered orally did not decrease weight gain or feed conversion. In such cases, the primary health status of the birds is important, as well as an adequate, non-limiting supply of nutrients to prevent a decline in productivity. In addition to the subclinical dose of APEC, another important consideration is that the birds were not challenged on the first day of their lives, but later, on d 15 or d 50. According to the literature, the youngest birds infected with APEC respond with reduced productivity, although a compensatory growth effect is often observed in later rearing stages (Chasser et al., 2021; Christensen et al., 2021). However, despite the fact that APEC infection had no influence on the growth performance of turkeys, its effects were noted in the air sac, and in the enzyme and metabolic activity of cecal microbiota. It was not our intention to induce an infection resulting in bird death, but only a subclinical infection which is frequently encountered in poultry production. Our previous study (Mikulski et al., 2022) revealed that the early short-term administration of antibiotics or continuous use of the coccidiostat monensin in clinically healthy turkeys (uninfected with APEC) had no effect on growth performance. Similarly, Charleston et al. (1998) reported that the chickens that survived initial IBV infection and intratracheal challenge of 10^6^ CFU of E. coli or that recovered after medication grew at a rate similar to that noted in uninfected birds between d 14 and 21 of the study. In a study of the effectiveness of antibiotics in the treatment of septicemia caused by triple APV/E.coli/ORT infection in 3-wk-old turkeys, no deterioration in their growth performance was observed, either (Marien et al., 2007).

The changes in ileal viscosity and DM, observed in both experiments, indicate that the effects of *E. coli* infection persist in the GIT for a longer period of time and/or are more pronounced when birds are exposed to the pathogen in an early stage of rearing. The increased viscosity of ileal digesta could result from a decrease in hydration, which in turn could be due to increased intestinal permeability that is often observed during *E. coli* infection (Chasser et al., 2021; Tejeda and Kim, 2021). Both early and late *E. coli* challenges resulted in increased hydration of the cecal digesta and increased ammonia concentration measured on d 56. Cecal ammonia levels were also elevated in younger infected birds on d 21. Although ammonia does not contribute significantly to an unfavorable increase in cecal pH, an increase in its concentration affects the health of the large intestine (Gugolek et al., 2020). In the current study, the *E. coli* challenge could impair small intestinal digestion by increasing protein bypass from the ileum to the ceca. As a result, cecal deamination reactions generate more ammonia than can be utilized by proliferating bacteria. According to Richardson et al. (2013), *Enterobacteriales* are able to grow on both peptides and amino acids, which leads to increased ammonia production.

Microorganisms play a key role in fermentation processes in the birds’ ceca (Wen et al., 2023). Before being absorbed by the microbial cell, complex macromolecules must first be hydrolyzed into small particles by extracellular enzymes. Therefore, extracellular enzymes are important variables influencing how organic matter is utilized in the large gut ecosystem (Ramin and Allison, 2019). In the present study, both early and late *E. coli* challenges resulted in enhanced extracellular activity of several bacterial enzymes, that is, β-glucosidase, α-galactosidase, α-arabinofuranosidase, and β-glucuronidase. β-Glucosidase is widespread among intestinal microbiota, and it may play an important role in the release of active (e.g., antioxidant) molecules (Murota et al., 2018). On the other hand, under specific conditions, microbial β-glucosidase may be responsible for an undesirable increase in toxin and xenobiotic uptake from the intestine into the bloodstream (Michlmayr and Kneifel, 2014). α-Galactosidase and α-arabinofuranosidase are exoglycosidases that targets branched polysaccharides and lignocellulosic plant cell wall components (Bhatia et al., 2020; Akkaya et al., 2023). By reversing the detoxifying process known as glucuronidation, microbial β-glucuronidase may hinder the excretion of substances from the body such as environmental toxins (Dashnyam et al., 2018). High activity of β-glucuronidase is associated with pathogenic bacterial species rather than beneficial ones (Juśkiewicz and Zduńczyk, 2002; Lee et al., 2022). According to the adopted research hypothesis, late infection induced a more evident increase in extracellular enzyme activities. Those findings corroborate the results noted in younger turkeys challenged on d 15 and slaughtered on d 21 as well as in older ones infected on d 50 and scarified on d 56. Such an increase points to significant mobilization of cecal microbiota for increased enzymatic digestion of substrates to obtain additional energy for optimal growth. As a result, the quantitative production of cecal SCFAs was not reduced.

The second main factor in the present experiments was the early administration of selected antibiotics, that is, monensin (administered throughout the entire rearing period, for 21 d or for 56 d in Experiments 1 and 2, respectively) as well as enrofloxacin and doxycycline (both administered for the first 5 d of feeding). Interestingly, in older birds, the continuous administration of monensin less considerably affected the enzyme activity of cecal microbiota and SCFAs production, compared with the other 2 antibiotics. In healthy uninfected turkeys, enrofloxacin and doxycycline, but not monensin, considerably reduced the cecal concentrations of SCFAs on d 56. In younger uninfected birds, the antibiotics did not affect the cecal concentrations of SCFAs on d 21. In Experiment 2, a substantial drop in the cecal concentrations of SCFAs was accompanied by diminished extracellular and/or total activities of cecal bacterial enzymes, i.e. α-glucosidase (enrofloxacin, doxycycline), β-glucosidase (doxycycline), α-galactosidase (enrofloxacin, doxycycline), and β-galactosidase (enrofloxacin, doxycycline). A similar tendency towards reduced effects on the enzyme activity of cecal microbiota was also observed in our previous study on growing turkeys subjected to enrofloxacin and doxycycline treatments (Mikulski et al., 2022). However, such a significant reduction in SCFA concentrations in response to enrofloxacin and doxycycline administration was not observed in the cited study. Those discrepancies should be further monitored. In a review article, Sakata (2019) listed a number of pitfalls in SCFA research and concluded that digesta SCFA concentration often does not adequately reflect the actual volume of their production. Sakata (2019) stressed the rapid absorption of SCFAs and suggested that their digesta concentration may vary even at different times of the day. Even considering the skepticism about SCFA concentration as a reliable parameter, it can be assumed that the early administration of enrofloxacin or doxycycline significantly affected cecal SCFA production and absorption in older turkeys on d 56. The available literature on the effects exerted by enrofloxacin, monensin, and doxycycline in the animals’ large gut focuses mainly on changes in microbial species composition but not on their metabolic activity, including SCFA production (Ma et al., 2020; Vieira et al., 2020; Greene et al., 2022). One of the few works investigating the link between dietary monensin application and cecal SCFA concentrations in broiler chickens revealed that they (in particular butyrate) increased in response to this coccidiostat (Kierończyk et al., 2020). In contrast, Boynton et al. (2017) and Greene et al. (2022) reported that even short-term administration of doxycycline in the early rearing of uninfected broiler chickens and in growing healthy mice caused long-term subsequent changes in the composition of cecal microbiota. Such an effect exerted by the tested antibiotics on microbiota in the cited studies as well as their potential impact on intestinal function could be responsible for the decrease in cecal SCFA levels observed in the current experiment.

The experimental antibiotic treatments applied to challenged birds exerted considerable modulatory effects on cecal digesta parameters. Older birds infected on d 15 and administered monensin and enrofloxacin had increased cecal DM concentration while those treated with enrofloxacin and doxycycline were characterized by increased cecal pH values. A significant drop in cecal ammonia concentration was noted in all older antibiotic-treated birds challenged in a later stage of rearing. The above changes can hardly be seen as positive or negative, since a decrease in toxic ammonia levels can be considered positive, whereas an increase in pH values is not beneficial to the cecal ecosystem. An analysis of the activities of individual bacterial enzymes, observed in the present study, clearly indicates that the early administration of antimicrobial agents, especially enrofloxacin and doxycycline, exerts long-term effects on the enzyme activity of cecal microbiota. In both experiments, birds challenged with APEC were characterized by enhanced extracellular and total activities of cecal bacterial enzymes and the administered antibiotics reduced those activities. In all infected birds, a key effect of antibiotic therapy was a decrease in the activity of bacterial β-glucuronidase, an enzyme characteristic of pathogenic bacteria (Bhatt et al., 2020). The activity of β-glucuronidase is not specific only for *E. coli,* and it also found in *Shigella* and *Yersinia* strains, as well as *Flavobacterium* spp., *Bacteroides* spp., *Staphylococcus* spp., *Streptococcus* spp., and *Clostridium* spp. (Fiksdal and Tryland, 2008). In older turkeys (Experiment 2), the applied treatments had no effect on cecal SCFA concentrations. This finding is very interesting taking into account significant variations in the enzyme activity of cecal microbiota in birds infected with *E. coli* and/or treated with antibiotics. Such results indicate that cecal microbiota have high adaptive capacity and are able to maintain adequate levels of SCFA production. In younger turkeys slaughtered on d 21, SCFA concentrations were highest in infected birds treated with doxycycline but at the same time those turkeys had the highest ammonia levels and cecal pH values. These results do not support previous findings (Gugolek et al., 2020) which have suggested that SCFA concentrations and not ammonia levels exert the greatest influence on the resultant pH of intestinal digesta. This issue should therefore be revisited, as both SCFAs and ammonia as well as the acidity of intestinal digesta have a profound impact on the health status of the large intestine and gut microbiota.

As indicated above, birds tolerated the subclinical infection process quite well, and it did not adversely affect their productivity. However, the experimental factors (antibiotics, challenge) had a significant effect on fermentation processes and the activity of cecal microbial enzymes. Nevertheless, the diets fed to turkeys provided them with nutritional comfort, and the digestive processes in the small intestine were not so disturbed as to result in a slower growth rate. Our previous studies investigating microbial processes in the avian large intestine confirmed that SCFAs support nutrient utilization, thus promoting the growth of birds, in particular when diets contained less-digestible dietary fractions, e. nonstarch polysaccharides or fiber-polyphenol complexes (Zduńczyk et al., 2014; 2016). In the present experiment, the experimental factors were not diet-related and the diets did not contain anti-nutritional components. This does not change the fact that the health status of the ceca, including enzyme activity and microbial metabolite profile, is of paramount importance in the production of premium-quality poultry products (Przywitowski et al., 2017). Therefore, this study focused on intestinal processes that are often disregarded by researchers analyzing the growth performance and productivity of birds.

## CONCLUSIONS

Both experiments demonstrated that both early and late APEC challenges contributed to an increase in ammonia levels in the cecal digesta and hydration as well as ileal pH values. In addition, the early APEC challenge increased ileal DM concentration and viscosity in older turkeys. The above findings indicate that the effects of *E. coli* infection persist in the GIT for a longer period of time and/or are more pronounced when birds are exposed to the pathogen in an early stage of rearing. In both experiments, the *E. coli* challenge enhanced the extracellular activity of several cecal bacterial enzymes, that is, β-glucosidase and β-glucuronidase, especially in older birds infected with APEC in a later stage of life. These birds had higher release rates of most analyzed enzymes from bacterial cells into the cecal digesta milieu. In the unfriendly APEC environment, such mechanisms could contribute to more efficient cecal harvest of energy from undigested nutrients, thus promoting undisturbed production of SCFAs by cecal microbial communities. The continuous administration of monensin throughout the rearing period resulted in a weaker gastrointestinal response in older birds, compared with the other 2 antibiotics administered only for the first 5 d of feeding. In conclusion, the challenge with APEC as well as the administration of the antimicrobials enrofloxacin and doxycycline exerted strong modulatory effects on the activity of cecal microbiota. Unfortunately, the results of the study are not conclusive as both desirable and undesirable effects of preventive early short-term antibiotic therapy were observed in turkeys, including normalization of ileal viscosity and cecal ammonia concentration, and disruption in cecal SCFA production.

## Appendix Supplementary materials

Image, application 1

## Footnotes

Supplementary material associated with this article can be found in the online version at doi:10.1016/j.psj.2024.103720.
