# Supplementary material for: Gastrointestinal response to the early administration of antimicrobial agents in growing turkeys infected with Escherichia coli
Source: Poult Sci. 2024 Apr 3;103(6):103720. doi: 10.1016/j.psj.2024.103720 (PMC11063517; doi:10.1016/j.psj.2024.103720)
Supplement: Supplementary file 1 [file mmc1.docx]

**Supplementary Table 1.** Ingredient composition and nutrient content of turkey diets.

| g/100 g, as-fed basis | Feeding period, days | |
| --- | --- | --- |
|  | d 0 to 28 | d 29 to 56 |
| Ingredients |  |  |
| Wheat | 48.50 | 56.26 |
| Soybean meal (46% CP) | 42.32 | 33.27 |
| Rapeseed meal, full fat (20.7% CP) | 2.00 | 4.00 |
| Soybean oil | 2.01 | 1.88 |
| Sodium bicarbonate | 0.15 | 0.15 |
| Sodium chloride | 0.19 | 0.2 |
| Limestone | 1.44 | 1.43 |
| Monocalcium phosphate | 1.84 | 1.51 |
| L-Lysine HCL | 0.54 | 0.47 |
| DL-Methionine | 0.32 | 0.21 |
| L-Threonine | 0.16 | 0.09 |
| Ronozyme P | 0.01 | 0.01 |
| Ronozyme WX | 0.02 | 0.02 |
| Vitamin-mineral premix^1^ | 0.50 | 0.50 |
| Calculated nutrient content |  |  |
| Metabolizable energy, kcal/kg | 2750 | 2850 |
| Crude protein | 26.50 | 23.50 |
| Lysine total | 1.75 | 1.50 |
| Methionine + Cysteine total | 1.12 | 0.95 |
| Threonine total | 1.08 | 0.90 |
| Calcium | 1.20 | 1.10 |
| Available phosphorus | 0.58 | 0.50 |
| Na | 0.14 | 0.14 |

^1^ Provided per kg diet (feeding periods: d 0 to 28 and d 29 to 56): mg: retinol 3.78 and 3.38, cholecalciferol 0.13 and 0.12, α-tocopheryl acetate 100 and 90, vit. K_3_ 5.8 and 5.6, thiamine 5.4 and 4.7, riboﬂavin 8.4 and 7.5, pyridoxine 6.4 and 5.6, cobalamin 0.032 and 0.028, biotin 0.32 and 0.28, pantothenic acid 28 and 24, nicotinic acid 84 and 75, folic acid 3.2 and 2.8, Fe 64 and 60, Mn 120 and 112, Zn 110 and 103, Cu 23 and 19, I 3.2 and 2.8, Se 0.30 and 0.28, respectively.

**Supplementary Table 2****.** Body weight and body weight gain of turkeys in Experiment 1.

|  | Body weight (kg) | | Body weight gain (kg) | |
| --- | --- | --- | --- | --- |
|  | d 15 | d 21 | d 0 to 15 | d 15 to 21 |
| Antibiotic (A)^1^ |  |  |  |  |
| C | 0.355 | 0.614 | 0.298 | 0.259 |
| M | 0.348 | 0.602 | 0.291 | 0.254 |
| E | 0.350 | 0.606 | 0.293 | 0.255 |
| D | 0.341 | 0.589 | 0.284 | 0.248 |
| Challenge (Ch)^2^ |  |  |  |  |
| - | 0.355 | 0.614 | 0.298 | 0.259 |
| + | 0.342 | 0.591 | 0.285 | 0.249 |
| Interaction (A × Ch) |  |  |  |  |
| C- | 0.356 | 0.616 | 0.299 | 0.260 |
| C+ | 0.354 | 0.612 | 0.297 | 0.258 |
| M- | 0.359 | 0.620 | 0.302 | 0.261 |
| M+ | 0.337 | 0.583 | 0.280 | 0.246 |
| E- | 0.351 | 0.606 | 0.294 | 0.255 |
| E+ | 0.350 | 0.605 | 0.293 | 0.255 |
| D- | 0.356 | 0.615 | 0.299 | 0.259 |
| D+ | 0.326 | 0.563 | 0.269 | 0.237 |
| SEM | *0.004* | *0.007* | *0.004* | *0.003* |
| *P*-value |  |  |  |  |
| A | *0.708* | *0.708* | *0.707* | *0.709* |
| Ch | *0.128* | *0.127* | *0.128* | *0.127* |
| A × Ch | *0.566* | *0.568* | *0.566* | *0.570* |

^1^ C, untreated control; M, treated with monensin (90 mg per kg of feed, for 21 d); E, treated with enrofloxacin (10 mg per kg of BW, added to drinking water for five consecutive days after hatching); D, treated with doxycycline (at a dose of 50 mg per kg of BW, added to drinking water for five consecutive days after hatching).

^2^ On d 15, birds were challenged with avian pathogenic *E. coli* (+) or served as an uninfected control group with no challenge (-).

The average initial d 0 BW of poults was 56.9 g (SD = 0.254).

**Supplementary Table 3.** Body weight and body weight gain of turkeys in Experiment 2.

|  | Body weight (kg) | | Body weight gain (kg) | |
| --- | --- | --- | --- | --- |
|  | d 50 | d 56 | d 0 to 50 | d 50 to 56 |
| Antibiotic (A)^1^ |  |  |  |  |
| C | 2.58 | 3.31 | 2.52 | 0.74 |
| M | 2.68 | 3.45 | 2.63 | 0.77 |
| E | 2.52 | 3.24 | 2.46 | 0.72 |
| D | 2.57 | 3.32 | 2.52 | 0.74 |
| Challenge (Ch)^2^ |  |  |  |  |
| N | 2.55 | 3.28 | 2.49 | 0.73 |
| R | 2.60 | 3.35 | 2.54 | 0.75 |
| L | 2.62 | 3.36 | 2.56 | 0.75 |
| Interaction (A × Ch) |  |  |  |  |
| CN | 2.59 | 3.34 | 2.54 | 0.74 |
| CR | 2.51 | 3.23 | 2.46 | 0.72 |
| CL | 2.62 | 3.37 | 2.57 | 0.75 |
| MN | 2.56 | 3.29 | 2.50 | 0.73 |
| MR | 2.79 | 3.59 | 2.73 | 0.80 |
| ML | 2.70 | 3.47 | 2.64 | 0.77 |
| EN | 2.49 | 3.20 | 2.43 | 0.71 |
| ER | 2.65 | 3.41 | 2.59 | 0.76 |
| EL | 2.42 | 3.12 | 2.37 | 0.70 |
| DN | 2.56 | 3.30 | 2.50 | 0.74 |
| DR | 2.45 | 3.16 | 2.39 | 0.71 |
| DL | 2.71 | 3.49 | 2.66 | 0.78 |
| SEM | *0.032* | *0.041* | *0.032* | *0.009* |
| *P*-value |  |  |  |  |
| A | *0.330* | *0.331* | *0.330* | *0.330* |
| Ch | *0.685* | *0.690* | *0.685* | *0.706* |
| A × Ch interaction | *0.331* | *0.345* | *0.331* | *0.397* |

^1^ C, untreated control; M, treated with monensin (90 mg per kg of feed, for 56 d); E, treated with enrofloxacin (10 mg per kg of BW, added to drinking water for five consecutive days after hatching); D, treated with doxycycline (at a dose of 50 mg per kg of BW, added to drinking water for five consecutive days after hatching).

^2^ On d 15 (R) or d 50 (L), birds were challenged with avian pathogenic *E. coli* or served as a control group with no challenge (N).

The average initial d 0 BW of poults was 56.9 g (SD = 0.254).
